# Parental age and developmental milestones: pilot study indicated a role in understanding ADHD severity in Indian probands

**DOI:** 10.1186/s12887-019-1483-x

**Published:** 2019-04-22

**Authors:** Subhamita Maitra, Kanchan Mukhopadhyay

**Affiliations:** 1 Manovikas Biomedical Research and Diagnostic Centre, Manovikas Kendra, 482, Madudah, Plot I-24, Sec.-J, E.M. Bypass, Kolkata, 700107 India; 2 Present address: Mahidol University, Salaya, Thailand

**Keywords:** ADHD, Birth history, Developmental milestone, Behavioral problem, Inattention, Impulsivity

## Abstract

**Background:**

In different ethnic groups, birth related factors have shown significant influence in the etiology of Attention deficit hyperactivity disorder (ADHD). Based on these interesting findings, we aimed to investigate association between different pre- and post natal variables and ADHD associated traits in Indian subjects.

**Methods:**

ADHD Probands recruited based on the DSM-IV, were assessed by the Conner’s Parent Rating Scale for behavioral problem (BPr), inattention (IA), hyperactivity (HA) and ADHD index (AI). Impulsivity (Imp) was assessed by the Tsukuyama scale.

**Results:**

Higher paternal (Std β = 0.23) and lower maternal (Std β = 0.21) age showed significant association with Imp of the probands. Higher paternal age also revealed association with BPr (Std β = 0.18). Age of onset was distinctly associated with AI (Std β < 0.16) while developmental delay was negatively correlated with BPr, Imp, IA and birth weight (r < − 0.13); also confirmed by Posthoc-ANOVA (*P* < 0.05).

**Conclusion:**

We infer that parental age, developmental delay and birth related variables may have a cumulative effect on ADHD symptom severity.

**Electronic supplementary material:**

The online version of this article (10.1186/s12887-019-1483-x) contains supplementary material, which is available to authorized users.

## Background

Attention Deficit Hyperactivity Disorder (ADHD) is a neuro-developmental disorder, mostly detected in school-going children/adolescents [1, 2]. Apart from the cardinal symptoms of age inappropriate inattention (IA), hyperactivity (HA) and impulsivity (Imp), behavioral problem (BPr) and cognitive deficit are major traits in ADHD [3, 4]. A number of co-morbid conditions like oppositional defiant disorder, conduct disorder, mood disorder, learning difficulties, etc. are also often detected in ADHD probands [5] making accurate diagnosis even more difficult.

Being a multigenic disorder, disease etiology is influenced by complex interplay between biological and environmental factors. As compared to age matched healthy children, ADHD probands often suffer from delayed maturation of the brain [6, 7]. Maternal health during pregnancy, along with genetic and familial factors, was suggested as a variable for disease susceptibility as well as symptom severity [8]. Maternal thyroid dysfunction [9] and mental health issues also showed association with disease onset in later life. A meta-analytic study indicated almost double ADHD frequency, impaired cognitive functions, learning problems, behavioral outburst among preterm children [10]. Investigators further claimed a role of parental age in disease susceptibility [11–16]. Maternal age, birth order, peri-natal complication, and parenting, were also speculated to add up to the disease incidence [17–19]. Perinatal hypoxia, crucial factor for the well being of neurons and dopaminergic cells, was also suggested to have a primary role in ADHD [20]. Though a few researchers have explored the role of parental influences on the occurrence as well as severity of ADHD, a high range of inconsistency has been portrayed.

Several reports show that prevalence of ADHD is increasing in India [21–25]. However, in spite of appreciating a possible role of birth related variables in disease penetrance, reports from India are little scanty. Based on this lacuna, we investigated whether any pre- or post-natal factor has any influence on ADHD associated symptoms in this ethnic group.

## Methods

### Demographic variables

ADHD probands (*N* = 212), age 6–14 years (M:F 189:23) were recruited following the Diagnostic and Statistical Manual-IV- Text Revised (DSM-IV TR) [26] and details of birth history as well as developmental milestones were recorded. Majority of the probands belonged to the combined subtype (73%), while only a small number of probands were predominantly HA (12%) and IA (15%) subtypes.

BPr, IA, and HA was assessed by DSM-IV-TR [26] and Conner’s Parent Rating Scale-Revised (CPRS-R) [27]. Impulsivity associated with interpersonal relations (Int_Per_Imp) and school work (Schl_Work_Imp) was assessed through Domain Specific Impulsivity Scale [28]. Age of onset was considered as the age when symptoms were first noticed. The study was approved by the Institutional Human Ethical committee.

### Inclusion/exclusion criteria

Children diagnosed primarily with ADHD were considered for the study. Those with only psychiatric problems including pervasive developmental disorders, any form of mental retardation (IQ ≤ 70) and fragile-X syndrome, were excluded.

### Score conversion

Order of the child was scored as 1 for the 1st child, 2 for the 2nd child and likewise. Probands with no developmental delay were grouped under 1 while those with only delayed speech was considered as 2 and with delay in motor as well as other milestones as 3. Probands delivered normally were scored as 1 while probands delivered by caesarean section and forcep’s were scored as 2 and 3 respectively. Preterm delivery was scored as 1, full term delivery was scored as 2 and post-term deliveries were considered as 3.

### Statistical analyses

All analysis was done using the SPSS version 32. Each and every necessary assumption was checked before running the appropriate program. Principal Component analysis (PCA) was performed for the entire dataset excepting for nominal predictors like Term and Order which had low number of individuals in the third group. Initial check for sphericity and adequate sampling were done for the total dataset.

## Results

### Details on birth related parameters

**Fig. 1:**
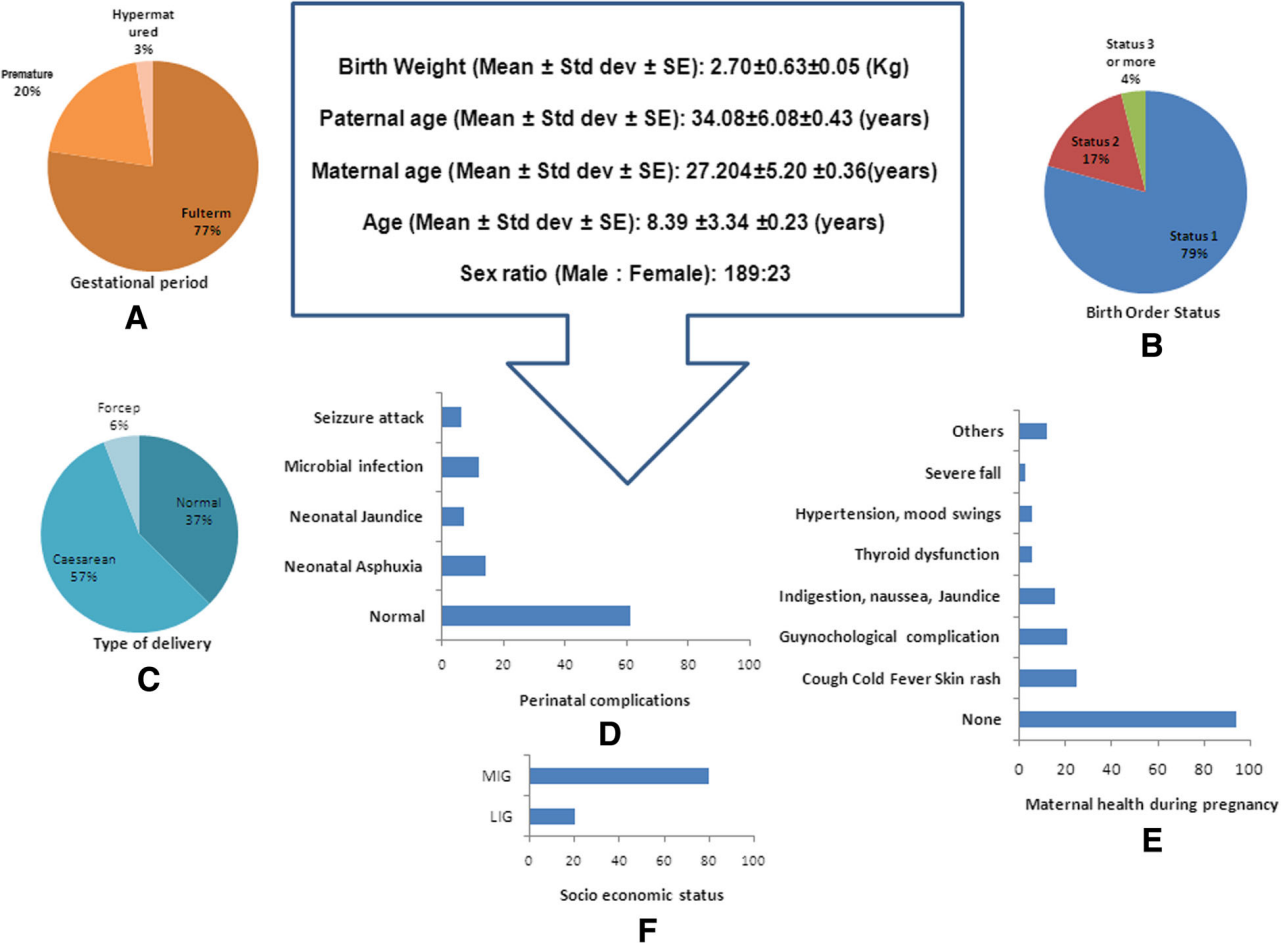
Socio demographic variables observed in the studied population

Mean age of the probands was (8.39 ± 3.34). Average paternal age (P_Age) was 34 years (34.14 ± 6.19) while maternal age (M_Age) was 27 years (27.04 ± 5.26). Most of the probands were delivered at full term, while 20% were premature and only few were hypermatured (Fig. 1a). Analysis on birth order (Order) showed that maximum probands were the first child while second and third issues were few (Fig. 1b) and Caesarean section (C_S) was the commonest form of delivery (Fig. 1c). Only few children suffered from neonatal problems (Fig. 1d). Most of the mothers’ maintained good health while few had gestational complications of hypertension, mood swings, indigestion, cold allergy, jaundice, thyroid dysfunction (Fig. 1e). Probands mostly belonged to middle to higher income groups (Fig. 1f).

### Principal component analysis

**Fig. 2:**
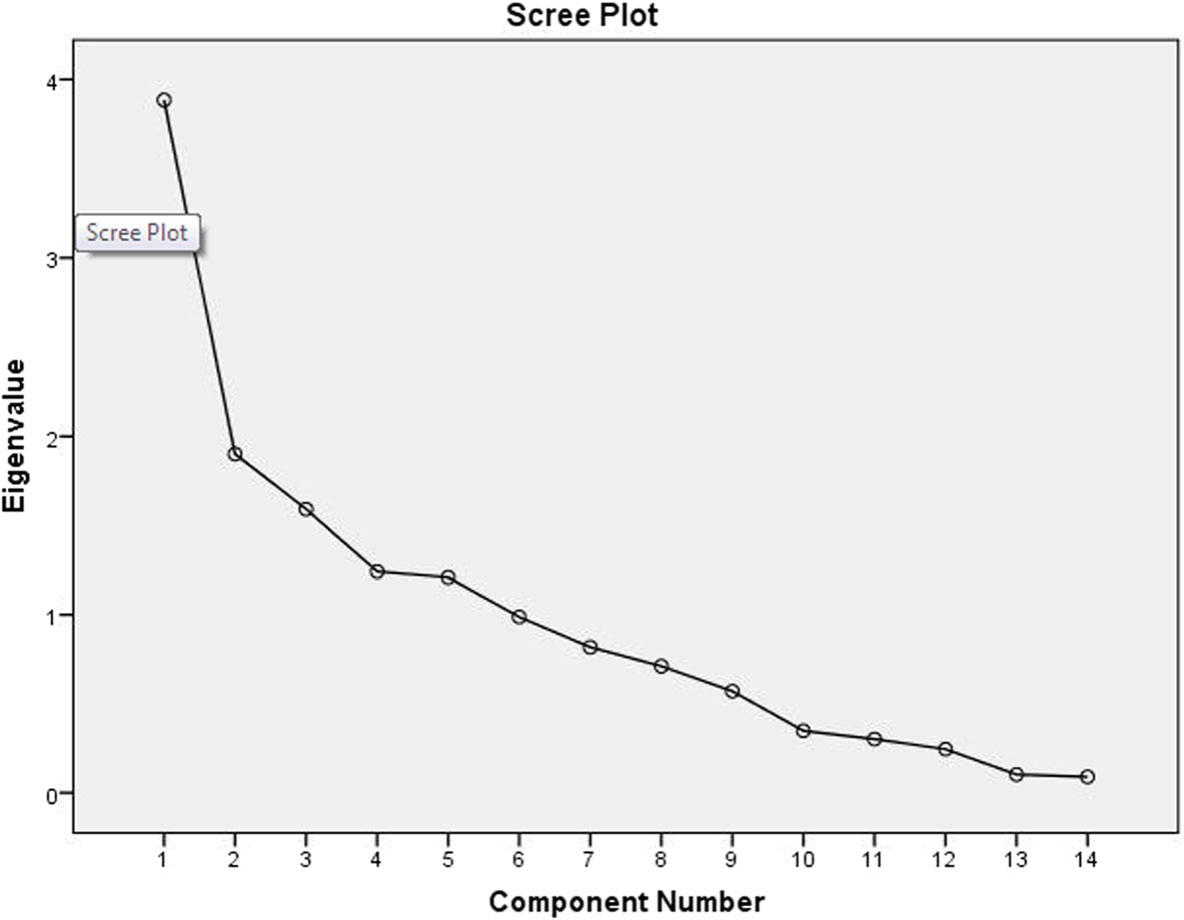
Scree plot of eigen value for corresponding component

Test for sphericity revealed significant insight for the dataset (*P* < 0.01). However, sampling adequacy was low (KMO = 0.643) and hence, a preliminary analysis was performed using a varimax rotation model. The extracted communality values showed percentage of variance that could be explained by the corresponding model (Table 1). More than 60% of the variance could be explained for all the variables excepting for birth weight (B_weight) and disease onset (Onset) (Table 1). Component matrix analysis extracted the traits into 5 components (Table 2). Out of the five, the first Component included major contributions of CPRS_AI, CPRS_BPr, Int_Per_Imp, CPRS_IA, DSM_IA, Schl_Work_Imp, DSM_HA, CPRS_HA, DSM_Imp. Parental age and development (Milestones) were significant under Component 2 and 4 (Table 2). Component 4 showed moderate effects of Milestone, DSM_HA, CPRS_HA, DSM_Imp, Onset, B_weight while Component 5 exhibited effects of traits and Milestones (Table 2). After varimax rotation, Components were displaced further (Table 3). The primary component 1 included Schl_Work_Imp, DSM_IA, CPRS_IA, CPRS_AI, while parental age was included under Component 4 only (Table 3). Onset and Milestones came under Component 3 and Component 5 respectively (Table 3). Analysis of variance caused by each component showed more than 50% contribution by the first three Components (Additional file 1: Table S1; 27.7, 13.57, 11.36 respectively). The Scree Plot (Fig. 2) showed highest eigen value for Component 1 with higher eigen values for the 2nd and 3rd Component than the rest. Thus, it is apparent that variables involved in the first three Components are significant points for the present dataset.

**Table 1:**
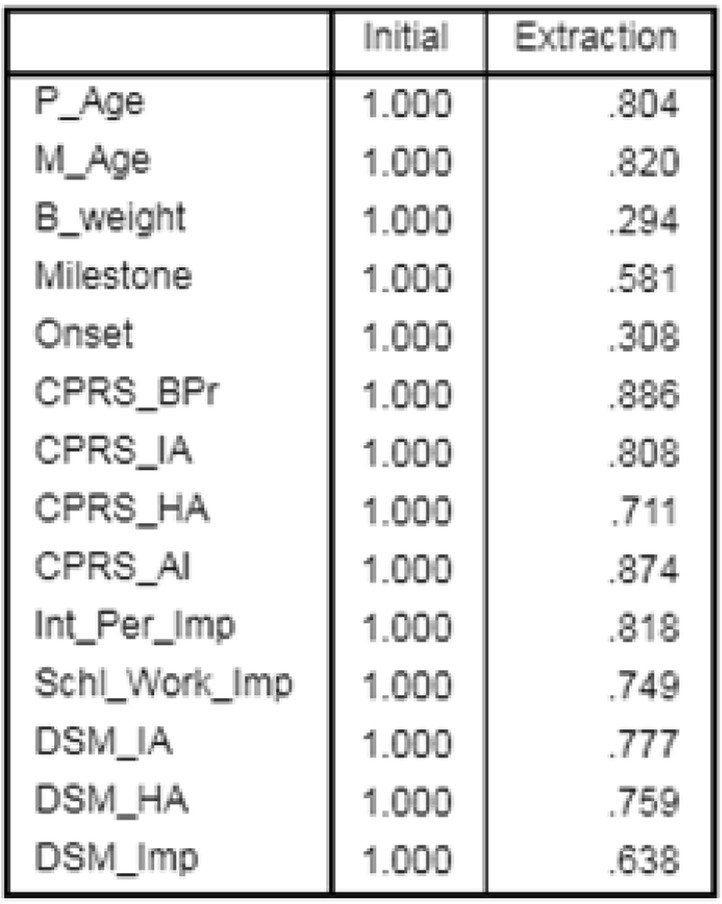
Communalities of the variables obtained from the Varimax rotation model

**Table 2:**
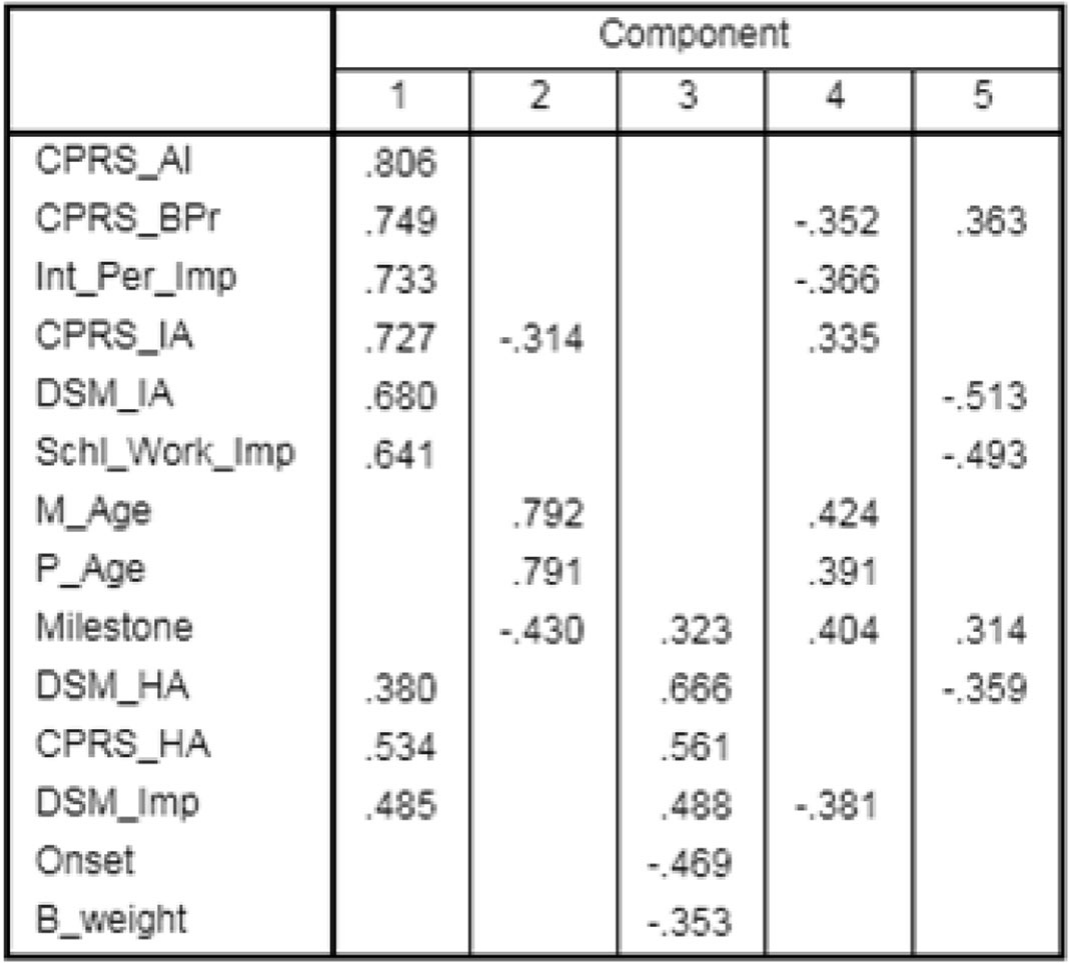
Initial correlation observed between components and its corresponding variables; rotation converged in 9 iterations

**Table 3:** Component matrix after Varimax rotation

Variables	Rotated Component Matrix^a^				
	1	2	3	4	5
Schl_Work_Imp	0.816				
DSM_IA	0.796				
CPRS_IA	0.778	0.353			
CPRS_AI	0.753	0.473			
CPRS_BPr		0.903			
Int_Per-Imp		0.857			
DSM_HA			0.856		
DSM_Imp		0.394	0.683		
Onset			0.388		
M_Age				0.896	
P_Age				0.885	
Milestone					0.718
CPRS_HA		0.357	0.457		0.557
B_weight					−0.509

Extraction Method: Principal Component Analysis; Rotation Method: Varimax with Kaiser Normalization; a. Rotation converged in 9 iterations

### Linear correlation analysis

Continuous variables were analyzed by Pearson’s correlation test and the ordinal variables were analyzed by Spearman Rank correlation test (significant correlations are given in Additional file 1: Table S2). Positive correlations were found for P_Age-M_age (R = 0.66), P_Age-BPr (0.14), Order-P_Age (0.23), Order-M_Age (0.33). Onset was negatively correlated to DSM-HA (− 0.19) and CPRS-HA (− 0.16). Delivery was positively correlated with M_Age, B_weight, and Onset (R > 0.13). DSM-HA showed negative correlation with delivery (R-0.15). Developmental milestones were negatively correlated to B_weight, CPRS_BPr, Int_Per_Imp, Schl_Work_Imp (R > 0.13).

### Multiple regression analysis

Based on the nine outcome variables, nine different models were analyzed (Additional file 1: Table S3). Each of the models showed independence in observation in absence of multi-co linearity (Dabrin Watson [DW] 1.5–2.5, 1 < VIF < 10). Preliminary analysis was done including all the predictors (Term, Order, P_age, B_weight, Delivery, Milestone and Onset). But small number of subjects in the third category of Term, Order and Delivery (frequency < 0.10) made the models statistically weaker and these predictors were eliminated in the final step. Significant models were obtained for CPRS-BPr (*P* = 0.006) and Int_Per-Imp (*P* = 0.02). Though the overall model was not significant for DSM-AI and Schl_Work_Imp (*P* > 0.10), it was considered further due to closely significant T scores (Additional file 1: Table S4; P 0.08–0.007). Positive influence of P_Age was observed on higher BPr (*P* = 0.08). Higher P_Age and lower M_Age showed association with higher Int_Per_Imp (P = 0.02 & 0.04 respectively). AI was positively influenced by higher age of onset (*P* = 0.06). BPr, Int_Per_Imp and Schl_Work_Imp showed negative correlation with developmental milestones (*P* = 0.08–0.007). No other models showed any significant correlation.

### T test and ANOVA

Lower scores for BPr (P = 0.02) and AI (*P* = 0.052) were noticed in the preterm as compared to full term children (Table 4). Normally developing probands exhibited higher score for BPr, Int_Per_Imp (8.02 ± 3.22) and Schl_ Work_Imp (6.77 ± 2.87) as compared to those exhibiting developmental delay (Table 4). Values remained unaltered even after post-hoc analysis following ANOVA (*P* < 0.05).

**Table 4:** Comparative analysis on means

Symptom	Variable	Mean ± SDev	T test P	Posthoc P
BPr	Pre Term	6.35 ± 5.16	0.02	NA
	Full term	8.80 ± 5.36		
AI	Pre Term	23.33 ± 7.73	0.052	NA
	Full term	25.96 ± 6.34		
BPr	Normal	9.32 ± 5.45	0.0001	0.001
	Global	5.74 ± 4.91		
Int_Per_Imp	Normal	8.02 ± 3.22	0.004	0.01
	Global	6.45 ± 2.30		
Schl_Work_Imp	Normal	6.77 ± 2.87	0.02	0.056
	Global	5.67 ± 2.74		
DSM_IA	Normal	12.10 ± 4.73	0.064	0.14
	Global	10.63 ± 4.24		
AI	Normal	25.01 ± 6.29	0.065	0.17
	Speech	27.71 ± 6.66		

## Discussion

Decades of research has established a firm neuro-psychological basis for ADHD, though the actual remediation is still far away. Two central neuro-psychological models were hypothesized [1]. The first one is the executive or top down model indicating ADHD as a disorder of executive dysfunction affecting goal directed self organized flexible actions. The other theory is bottom up sensory/reward theory which suggest that ADHD patients lack motivation, reward and emotional regulation [1]. A combination of these two models gave rise to an integrated model portraying a complex clinical scenario. However, ADHD symptoms are highly heterogeneous and every domain of impairment may add up to a new level of complexity, making proposition of any individual model difficult [29–31].

**Fig. 3:**
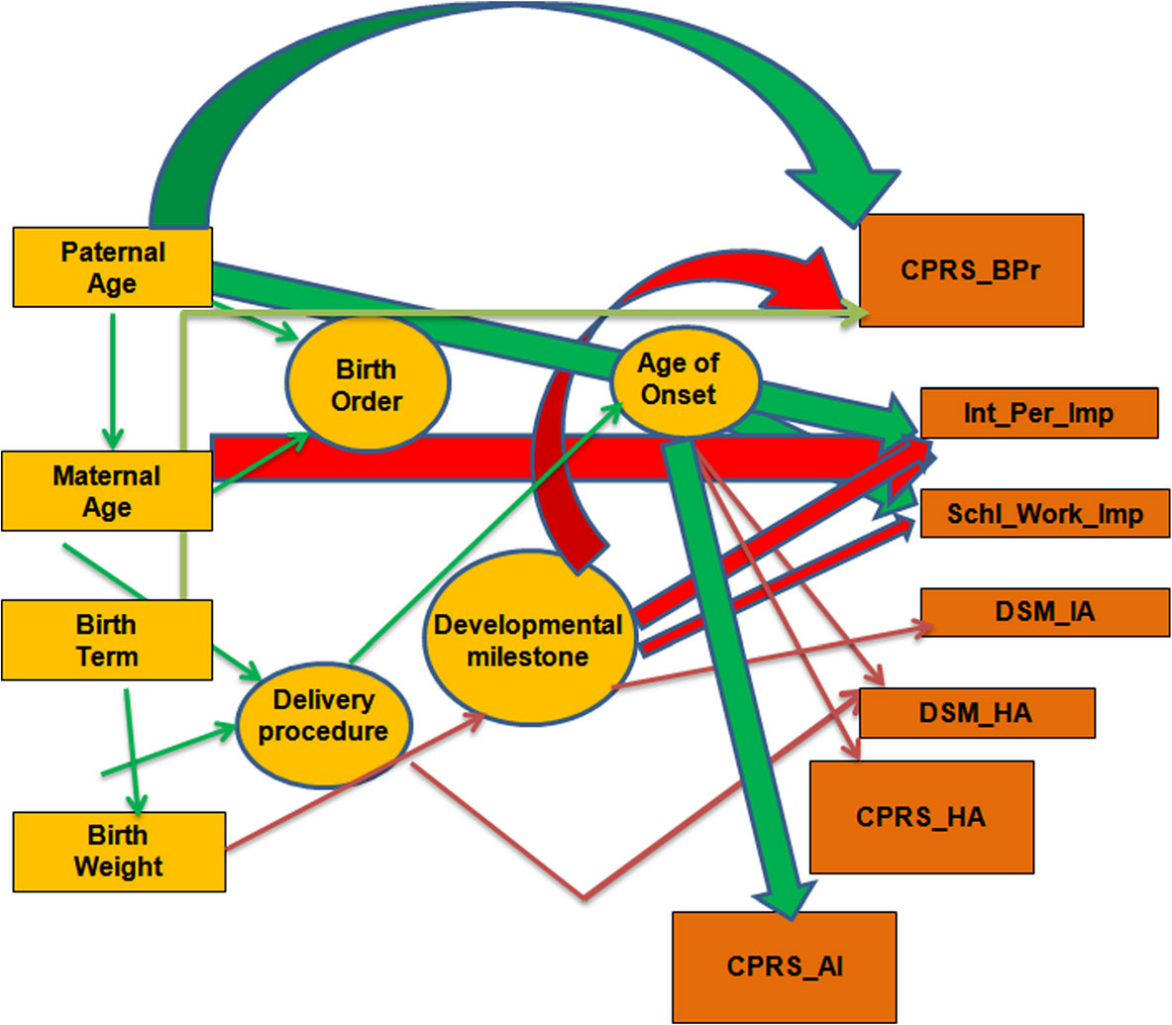
Manual path diagram to understand relation between different variables

In India, prevalence of ADHD was reported to be 11.33% among primary school children [22], 7.2% among adolescents [23], and 5.48% among college students [24]. However, detailed report on birth histories and their possible influence on the symptom severity were less frequently studied. Here, we report for the first time a role of birth related factors in the symptom severity of Indian ADHD probands. Significant influence of parental age, B_weight, delivery process, age of onset and developmental milestones were noticed on the trait scores. Based on the significant correlations detected amongst the predictable variables, we present a schematic diagram to understand the trajectory (Fig. 3). Parental age was identified as the principal independent factor. B_weight and term of delivery were not influenced by parental age, though the two were correlated amongst themselves. Thus, we have put birth term as another primary variable along with B_weight. After this primary level, three intermediate levels became evident. Birth order, being influenced by parental age was placed as the first intermediate variable. Process of delivery was the next in the same level; it was related to M_Age and B_weight. Milestone was also placed in the same level as it was influenced by the primary predictor B_weight. Since age of onset was only related to the delivery procedure, it was placed at the next intermediate level. Finally multiple regressions led to the isolation of multi- influential predictors that modified the symptom scores. All the predictor variables excepting the birth order and term were found to influence the symptoms and so the path diagram originated from Parental age, and ended in different trait scores traversing through birth weight, birth order, delivery procedure, developmental milestones and age of onset.

Studies from the western countries revealed a relationship between lower maternal age and higher vulnerability to ADHD [12–14]. Investigators also proposed association of lower maternal age with symptom intensity [11–16] and parenting was suggested to be better by older mothers [32]. Association between higher oppositional behavior as well as HA /Imp score of ADHD probands and higher maternal age were also reported [15]. However, effect of paternal age was not evident in Danish patients [13]. Our analysis revealed negative influence of maternal age on the Imp of probands; individuals born to younger mothers were more impulsive. Furthermore, higher paternal age was related to higher scores for BPr and Imp. We assume that these inconsistencies in associations may result from age differences, variation in sample size or a confounded effect of socio-demographic variations warranting further investigation.

A recent study from the Finland suggested association between low Apgar score and C_S with higher risk of ADHD [33]. We found correlation between higher maternal age and Caesarean/forcep delivery, associated with lower HA. Whether this contradictory finding is due to the presence of an actual socio-demographic difference warrants further in depth analysis in large cohort.

In the Caucasoid population, birth weight was speculated as a prime risk factor for developing ADHD symptoms, primarily inattention [30, 34–36]. In the Indo-Caucasoid population, we have noticed substantial influence of B_weight on the delivery process and developmental milestones which were associated further with symptom scores; preterm children showed lower score for BPr/AI. It can be speculated that B_weight showing negative association with developmental delay could be a marker for overall development and along with various intermediate factors, may affect ADHD associated traits.

Earlier investigators reported delay in motor development of ADHD probands [37]. Our study group showed delayed motor development in 26% probands. However, interestingly it showed negative influence on BPr; children with developmental delay exhibited reduced Imp. In an MRI based study on the developmental chronology of healthy brain, it was found that the affect and reward processing circuitry involving amygdale and nucleus accumbens develop faster than the cognitive control domain [38]. Nucleus accumbens, important for emotional circuitry, also has a role in motor control. Thus, it may be hypothesized that developmental delay in these regions may interfere with affective behavior as well as motor response while balancing the delayed cortical maturation, as reported in ADHD earlier [6]. However, the observed effect of delayed motor development on reduced impulsivity may also be partly due to seeking professional help at an early age. Our earlier study showed higher HA in the late onset group [39]. The present study revealed influence of late onset on increased AI and gradual decrease in HA on a continuous scale, indicating necessity of further analysis in larger cohort.

## Conclusion

Bio-demographic factors may assist in understanding the disease more clearly thereby providing chances for better rehabilitation. In an earlier study, we have reported that ADHD probands sustain impairment in attention while organizational efficiency improves with time and those with learning difficulties exhibit even more scholastic backwardness [40]. Our current analysis on the same group of probands suggests that parental age, low birth weight, and delivery process may affect symptom severity of ADHD probands, a finding which could be useful for monitoring growth of a child leading to early intervention. However, our number of subjects was limited. Successful replication in larger cohort and proper attention to birth related/ developmental factors may widen a contemporary window to provide more efficient management of ADHD related symptom severity.

## Additional file

Additional file 1: **Table S1.** Total variance explained by the principal components in the total data set. **Table S2.** Analysis of Correlation between identified variables. **Table S3.** Summary of multiple regression analysis. **Table S4.** Details of multiple regression analysis. (DOCX 41 kb)

## Abbreviations

ADHD: Attention deficit hyperactivity disorder
AI: ADHD index
B_weight: Birth weight
BPr: Behavioral problem
C_S: Caesarian section
CPRS_BPr: Behavioral problem assessed by CPRS-R
CPRS-AI: ADHD index scored by CPRS-R
CPRS-HA: Hyperactivity scored by CPRS-R
CPRS-IA: Inattention scored by CPRS-R
CPRS-Imp: Impulsivity scored by CPRS-R
CPRS-R: Conners’ Parents rating scale revised
Delivery: Term of delivery
DSM-HA: Hyperactivity scored by DSM-IV-TR
DSM-IA: Inattention scored by DSM-IV-TR
DSM-Imp: Impulsivity scored by DSM-IV-TR
DSM-IV-TR: Diagnostic and Statistical Manual of Mental Disorders-4th edition-text revised
HA: Hyperactivity
IA: Inattention
Imp: Impulsivity
Int_Per_Imp: Interpersonal impulsivity
M_age: Maternal age
Milestones: Developmental milestones
Onset: ADHD symptom onset
Order: Birth order
P_Age: Paternal age
PCA: Principal component analysis
Schl_Work_Imp: School work impulsivity
