# Supplementary material for: Parental age and developmental milestones: pilot study indicated a role in understanding ADHD severity in Indian probands
Source: BMC Pediatr. 2019 Apr 22;19:117. doi: 10.1186/s12887-019-1483-x (PMC6475966; doi:10.1186/s12887-019-1483-x)
Supplement: Supplementary file 1 — Table S1. Total variance explained by the principal components in the total data set. Table S2. Analysis of Correlation between identified variables. Table S3. Summary of multiple regression analysis. Table S4. Details of multiple regression analysis. (DOCX 41 kb) [file 12887_2019_1483_MOESM1_ESM.docx]

Table S1. Total variance explained by the principal components in the total data set.


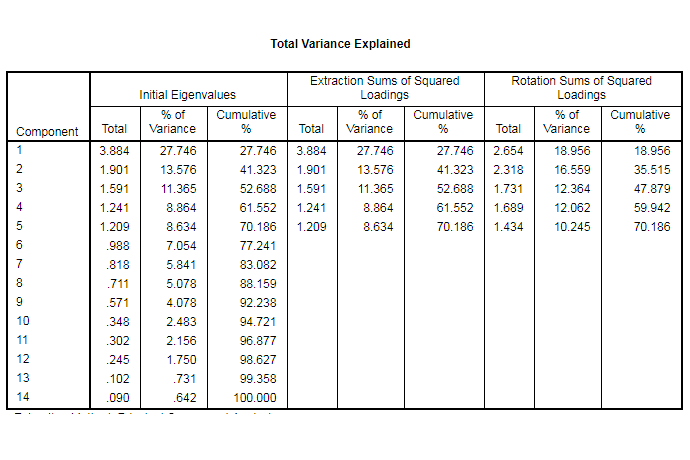


Table S2. Analysis of Correlation between identified variables.

| Variables | Correlation coefficient | P value | Type of test |
| --- | --- | --- | --- |
| P_Age-M_Age | 0.664 | <0.0001 | Pearson’s  correlation Test |
| P-Age-BPr | 0.139 | 0.072 |  |
| Onset-CPRS-HA | -0.19 | 0.01 |  |
| Onset-DSM-HA | -0.16 | 0.02 |  |
| Order-M_Age | 0.33 | <0.0001 | Spearman Rank  Correlation Test |
| Term-B_Weight | 0.33 | 0.0001 |  |
| Term-BPr | 0.18 | 0.02 |  |
| Delivery-M_Age | 0.27 | 0.001 |  |
| Delivery-B_Weight | 0.16 | 0.023 |  |
| Delivery-Onset | 0.13 | 0.065 |  |
| Delivery-DSM-HA | -0.15 | 0.035 |  |
| Milestone-B_Weight | -0.15 | 0.04 |  |
| Milestone-CPRS-BPr | -0.26 | 0.001 |  |
| Milestone-Int_Per_Imp | -0.18 | 0.01 |  |
| Milestone-Schl_Work_Imp | -0.15 | 0.04 |  |
| Milestone-DSM-IA | -0.13 | 0.08 |  |

Table S3. Summary of multiple regression analysis.

| Name of model | Multiple correlation  coefficient | F test score | P | DW Test | VIF |
| --- | --- | --- | --- | --- | --- |
| BPr | 0.32 | 3.39 | 0.006 | 1.69 | 1.10-1.83 |
| Int_Per-Imp | 0.28 | 2.88 | 0.016 | 1.48 | 1.09-1.86 |
| Schl-Work_Imp | 0.17 | 1.08 | 0.37 | 1.90 | 1.09 |
| AI | 0.17 | 0.85 | 0.52 | 1.81 | 1.03 |

Table S4. Details of multiple regression analysis

| Name of model | Variable | Standardized β | 95% I for β | T score (P) |
| --- | --- | --- | --- | --- |
| BPr | P_Age | 0.18 | -0.02 to 0.35 | 1.75 (0.08) |
|  | Milestone | -0.22 | -2.44 to -0.39 | -2.72 (0.007) |
| Int_Per_Imp | P_Age | 0.23 | 0.02 to 0.22 | 2.36 (0.02) |
|  | M_Age | -0.21 | -0.24 to -0.007 | -2.09 (0.04) |
|  | Milestone | -0.20 | -1.26 to -0.18 | -2.61 (0.01) |
| Schl_Work_Imp | Milestone | -0.14 | -0.94 to 0.06 | -1.75 (0.08) |
| AI | Onset | 0.16 | -0.01 to 0.72 | 1.90 (0.059) |
